# Immunostimulant Bathing Influences the Expression of Immune- and Metabolic-Related Genes in Atlantic Salmon Alevins

**DOI:** 10.3390/biology10100980

**Published:** 2021-09-29

**Authors:** Filipe Figueiredo, Harald Kristoffersen, Shripathi Bhat, Zuobing Zhang, Jacques Godfroid, Stefano Peruzzi, Kim Præbel, Roy Ambli Dalmo, Xiaoli Xu

**Affiliations:** 1 Norwegian College of Fishery Science, UiT—The Arctic University of Norway, N-9019 Tromsø, Norway; harald.kristoffersen@cermaq.com (H.K.); shripathi.bhat@uit.no (S.B.); kim.praebel@uit.no (K.P.); sscstgk@tj.gov.cn (X.X.); 2 College of Life Sciences, Shanxi University, Taiyuan 030006, China; zbzhang@sxu.edu.cn; 3 Department of Arctic and Marine Biology, UiT—The Arctic University of Norway, N-9019 Tromsø, Norway; jacques.godfroid@uit.no (J.G.); stefano.peruzzi@uit.no (S.P.)

**Keywords:** immunostimulants, innate immune system, pattern recognition receptors, gene expression, antiviral, metabolism, Atlantic salmon, alevins

## Abstract

**Simple Summary:**

Activation of immune cells is bioenergetically expensive, requiring precise control of cellular metabolism. This applies also to innate immune cells. The current study shows that the immunostimulants, Astragalus, Hyaluronic acid, Imiquimod, and Poly I:C can modulate the expression of genes involved in the innate antiviral immune, as well as genes associated with metabolism, in the early life stages of Atlantic salmon.

**Abstract:**

Disease resistance of fish larvae may be improved by bath treatment in water containing immunostimulants. Pattern recognition receptors, such as *TLR3*, *TLR7*, and *MDA5*, work as an “early warning” to induce intracellular signaling and facilitate an antiviral response. A single bath of newly hatched larvae, with Astragalus, upregulated the expression of *IFNα*, *IFNc*, *ISG15*, *MDA5*, *PKR*, *STAT1*, *TLR3,* and *TLR7* immune genes, on day 4 post treatment. Similar patterns were observed for Hyaluronic acid and Poly I:C. Increased expression was observed for *ISG15*, *MDA5*, *MX*, *STAT1*, *TLR3*, *TLR7*, and *RSAD2*, on day 9 for Imiquimod. Metabolic gene expression was stimulated on day 1 after immunostimulant bath in *ULK1, MYC, SLC2A1, HIF1A, MTOR*, and *SIX1*, in Astragalus, Hyaluronic acid, and Imiquimod. Expression of *NOS2* in Poly I:C was an average fourfold above that of control at the same timepoint. Throughout the remaining sampling days (2, 4, 9, 16, 32, and 45 days post immunostimulant bath), *NOS2* and *IL1B* were consistently overexpressed. In conclusion, the immunostimulants induced antiviral gene responses, indicating that a single bath at an early life stage could enable a more robust antiviral defense in fish. Additionally, it was demonstrated, based on gene expression data, that cell metabolism was perturbed, where several metabolic genes were co-regulated with innate antiviral genes.

## 1. Introduction

Atlantic salmon (*Salmo salar*) is, by far, the most economically important species in European aquaculture, particularly in Norway. A production of 1.4 million tonnes in 2019, valued at 6.6 billion EUR, represents almost 17% of gross domestic product (GDP) and makes Norway the second-largest producer in mariculture of finfish species in the world [1]. Among the many challenges that continuously afflict salmon production, viral diseases remain one of the most significant [2]. Building knowledge on the innate immunity of non-model organisms such as Atlantic salmon can contribute towards a better understanding of the fish immune and physiological responses to pathogens. The yolk sac period (without feeding) in Atlantic salmon is relatively prolonged, as alevins start feeding at approximately 300 day degrees (dd) after hatching. Throughout this period, the fish larva must rely mostly on innate immunity to protect themselves from infection [3]. 

At the core of the innate immune response lie two key families of pattern recognition receptors (PRRs): Toll-like receptors (TLRs) and retinoic acid-inducible gene-I-like receptors (RLRs) [4]. These receptors, whose existence was initially proposed by Charles Janeway Jr. [5], work as an “early warning system” to induce intracellular signaling and facilitate antiviral response. The evolutionarily ancient mechanism of recognizing pattern associated molecular patterns (PAMPs) and/or damage-associated molecular patterns (DAMPs) is responsible for triggering production cascades of cytokines such as interferon α (*IFNα*) and c (*IFNc*) through diverse pathways such as the MyD88-independent pathway, JAK-STAT and/or MAPK signaling pathways [6]. These cascades lead to the transcription and upregulation of IFN-stimulated antiviral genes such as interferon-stimulated gene 15 (*ISG15*), myxovirus resistance protein 1 (*MX1*), and radical S-adenosyl methionine domain-containing protein 2 (*RSAD2*; *viperin*), which potentially create a heightened antiviral state [7].

Stimulating the innate immune system of fish larvae may be a viable method to increase disease resistance [8], by leveraging its reaction to PAMPs. Immunostimulation of yolk-sac larvae may be achieved by bathing them in a solution containing immunostimulants—such as Poly I:C, Imiquimod, Hyaluronic acid, or other soluble substances. Thus, it is likely that substances that boost innate immunity may be beneficial to larvae by potentially increasing protection against pathogens.

Poly I:C (mimic of RNA virus) has been widely used to induce the expression of antiviral genes in fish [9,10]. It has been reported that Mrigal carp (*Cirrhinus mrigala*), bathed for 2 hr in water containing Poly I:C, expressed increased levels of MX transcripts [11]. While Poly I:C is widely used as a TLR3/TRIF pathway agonist [12], it can also induce a type I IFN response through the MDA5/NF-kB signaling pathway in miiuy croaker (*Miichthys miiuy*) [13]. Imiquimod was also responsible for antiviral gene induction in fish [14,15], as well as promoting gene expression via TLR7 sensing in mice [16], by activating the MyD88-dependent signaling pathway [16,17,18]. However, the effect of this substance has not been examined in fish larva after bath exposure. The same applies to Hyaluronic acid, which has been found to act as a damage-associated molecular pattern (DAMP), triggering sterile inflammation in mammalian animal models [19]. A study by Zhang et al. (2009) [20] showed that hyaluronic interfered with TLR4-dependent activation of Kupffer cells in vitro, while another experiment in a mouse model reported that bioactive hyaluronan suppressed the phosphorylation of several TLR4 signaling pathway proteins [21]. However, it is accepted that CD44 is the main receptor for Hyaluronic acid, which affects cell migration of, e.g., lymphocytes during inflammation. The transmembrane moiety of CD44 can be proteolytically cleaved yielding a 12 kDa intracellular domain that translocates to the nucleus and acts as a transcription factor, which, in turn, regulated the expression of genes involved in cell survival during stress and inflammation [22]. Hyaluronic acid has also been reported to capture/interact with viral particles, thus preventing the entry of viruses into cells [23]. CD44-like RNA sequences have been found in A. salmon (Acc. No: XM_014148324.1 and XM_01412512), but no further characterization has been performed.

One of the immunostimulants that has been extensively studied, by virtue of its ability to modulate immune mechanisms through gene upregulation, is the root extract from a plant belonging to the Astragalus genus (e.g., *Astragalus membranaceus*). Astragalus root is commonly used in traditional Chinese medicine. The modulatory effects of different Astragalus extracts have been demonstrated in fish species such as turbot (*Scophthalamus maximus*) [24], large yellow croaker (*Larimichthys crocea*) [25,26], Nile tilapia (*Oreochromis niloticus*) [27,28,29,30], largemouth bass (*Micropterus salmoides*) [31], grass carp (*Ctenopharyngodon idella*) [32], common carp (*Cyprinus carpio*) [33], yellow perch (*Perca flavescens*) [34], spotted maigre (*Nibea albiflora*) [35], yellow catfish (*Pelteobagrus fulvidraco*) [36], and Jian carp (*Cyprinus carpio* var. Jian) [37].

The antiviral properties of Astragalus root extract have also been demonstrated in vitro, during which it partly repressed replication of avian coronavirus (infectious bronchitis virus) in chicken embryo cells [38]. In another study, it was shown that Astragalus polysaccharides suppressed porcine circovirus type 2 replication by reducing oxidative stress and blocking the NF-kB pathway [39]. This response can be linked to the stimulatory effect Astragalus polysaccharides have on the TLR4-MyD88 dependent signaling pathway [40,41,42].

Activation of cells during, e.g., perturbation, may likely affect cell metabolism, and vice versa, as described in several reviews [43,44,45,46,47,48]. The accessibility of metabolites and nutrients may be challenging for immune cells throughout infection, owing to the altered local microenvironments that result from oxygen tension; particularly for innate immune cells such as macrophages [49].

When macrophages are activated by *danger signals*, the cells may undergo substantial changes with respect to metabolism to support cell growth, proliferation, functional transition and synthesis, and release of molecules. This requires metabolic adaptation to new microenvironments. Activated immune cells may have increased glycolytic activity (utilizing glucose, glutamine, and fatty acids to support the increased energy demand), reduced oxidative phosphorylation activity (hence reducing the formation of ATP), and modified tricarboxylic acid cycle (TCA) activity [50]. A heightened glycolytic activity is reminiscent of the Warburg effect [51]. Of the mentioned immunostimulants, only Poly I:C administration (injection) has been seen to significantly alter the metabolome of fish, as found in Chinook salmon (*Chinook tshawytscha*) and in yellow catfish (*Peiteobagrus fulvidraco*) [52].

The effects of Astragalus root extract, Hyaluronic acid, and Imiquimod after bath treatment in newly hatched Atlantic salmon alevins have never been examined before, neither have the effects of the selected immunostimulants on host gene coordinated expression of antiviral and metabolism genes. The current study is aimed at characterizing the influence of Astragalus, Hyaluronic acid, Imiquimod, and Poly I:C on the expression of genes involved in innate antiviral defense and metabolic processes.

## 2. Materials and Methods

### 2.1. Chemicals, Fish Stock, and Experimental Setup

Experimental groups were created with single batch Atlantic salmon alevins (402 days-degree eyed-ova provided by Benchmark Genetics, Bergen, Norway) at 530 day degrees. Similar numbers of alevins were distributed across 3 cylindrical incubators (50 L) per treatment (ca. 230–270 individuals/incubator), in a flow-through water system kept at an average temperature of 6–8 °C. One day after hatch, the alevins were bathed in 3 L glass beakers containing the immunostimulant (Table 1) (10 mg L^−1^) (1 mg L^−1^ for Imiquimod) for one hour, at 8 °C. The concentration was given as mg L^−1^ since the precise molecular weights of the immunostimulants were not well defined. The control group was not bathed and thus was not handled.

Water temperature was kept at 6–8 °C throughout the experimental period. The freshwater pH was 6.9. The average CaCO_3_ concentration in the inlet freshwater was 2.79 mg L^−1^ and Mg_2_^+^ 0.77 mg L^−1^. This means that the freshwater is very soft.

Following the exposure to the immunostimulant, alevins were returned to their respective incubators and sampled at 1-, 2-, 4-, 9-, 16-, 32-, and 45 days post exposure. At each sampling point, 36 alevins per treatment were euthanized with a lethal dose of benzocaine (Benzoak vet., 400 mg L^−1^; ACD Pharmaceuticals AS, Norway), placed in cryotubes containing RNAlater, and stored at −20 °C until RNA extraction.

Formal approval of the experimental protocol by the Norwegian Animal Research Authority (NARA) is not required since it falls under the purpose of recognized animal husbandry. These practices are exempt from the European convention on the protection of animals used for scientific purposes (EU Directive (2010/63/EU, cf. article 5d), which Norway has subscribed and implemented.

### 2.2. RNA Extraction and cDNA Synthesis

The Qiagen AllPrep RNA/DNA extraction mini kits were used (QIAGEN GmbH, Hilden, Germany) following the manufacturers’ specifications. The tail region, dorsal muscle, and yolk sac of the alevins at day 32 and 45 post hatch were removed before RNA isolation, while the yolk sac and tail region were removed from alevins at earlier time points, prior to homogenization. This resulted in the reduction in sample size and weight, yielding samples from 25 to 45 mg. Prior to extraction, 6 alevins per group from each sampling point, were homogenized (Precellys 24; Bertin Technologies, Montigny-le-Bretonneux, France), resulting in a total of 210 RNA samples. The purity and concentration of RNA were measured through Nanodrop (Thermo Fisher Scientific Inc., Waltham, MA, USA). In addition, gel electrophoresis was performed on approximately 50% of the samples to assess RNA integrity.

To synthesize the first-strand cDNA, the High-Capacity RNA-to-cDNA Kit (Applied Biosystems, Waltham, MA, USA) was used, with minor modifications to the manufacturer’s specifications. Samples and reaction mixture were incubated for 60 min at 37 °C, followed by a 5 min incubation at 95 °C to inactivate the reverse transcriptase. Samples were then stored at –20 °C until further processing. From an initial amount of ca. 2 µg of RNA, 20 μL of cDNA was synthesized.

### 2.3. qPCR

The qPCR reactions for each sample were run in duplicate, in an Applied Biosystems 7500 Fast Real-Time PCR System (Thermo Fisher Scientific Inc., Waltham, MA, USA). For each qPCR reaction, cDNA was diluted at 1:100, mixed with primers and Fast SyBr Green Mastermix (Thermo Fisher Scientific Inc., Waltham, MA, USA), and incubated according to the protocol detailed in Table 2.

The list in Table 3 was created by selecting primers from either previously published reports or unpublished results, based on their relevance to the immune system or metabolism of Atlantic salmon. Three housekeeping genes (HKG) were tested as potential references to normalize gene expression: *18S*, *β-actin*, and Elongation Factor 1 alpha (*EF1α*). From these three, *18S* was the most stable and consistent across a range of dilutions and hence was selected to serve as the endogenous control for this experiment.

### 2.4. Data Analysis

Results of qPCR in the form of threshold cycle values (*Ct*) were transformed into relative gene expression (*RGE*) between the gene of interest (*GOI*) and the *HKG*, using the *Pfaffl* method [62]. To achieve that, the converted primer efficiency (*E*) for each primer pair was calculated with the following equation:

$$E=\left(\frac{Primer\;efficency\;\left(\%\right)}{100}\right)\;+\;1$$

*RGE* was then calculated as follows:

$$RGE=\;\frac{{E(GOI)}^{\Delta Ct\left(GOI\right)}}{{E(HKG)}^{\Delta Ct\left(HKG\right)}}$$

where *ΔCt*(*GOI*) = Mean *Ct* Control-Mean *Ct* Sample, for the *GOI*, and *ΔCt*(*HKG*) = Mean *Ct* Control-Mean *Ct* Sample, for the *HKG*.

Data wrangling, statistical analysis and plotting was conducted in RStudio (version 1.4.1106, *TigerDaylily;* R version 4.0.3, *Bunny-Wunnies Freak Out*; RStudio Team, 2020). Packages used were: *ggpubr* [63], *grDevices* [64], *Hmisc* [65], *NCmisc* [66], *oaColors* [67], *patchwork* [68], *readxl* [69], rstatix [70], *stats* [64], *tidyverse* [71], and *utils* [64].

Data outliers were identified using the boxplot method from the *rstatix* R package. Only values above the third quartile-3xIQR (interquartile range), or below the first quartile-3xIQR, were removed. These are considered extreme outliers.

To determine which genes were significantly differentially expressed in relation to the control group, a *t*-test was used. For this, the mean expression of each gene, for each immunostimulant, was compared against the mean gene expression of a reference group (i.e., control) with the *compare_means* function of the *ggpubr* R package. Gene expression data are reported as mean ± standard error of the mean (SE). Genes were grouped by family to facilitate visualization. The correlation between immune and metabolic genes was calculated using a matrix of Spearman’s rank correlation coefficients. This method allowed for gene expression values to be correlated throughout all sampling days.

Significant statistical differences are shown in plots when relevant (*p <* 0.05), in the form of an asterisk. All *p*-values were adjusted using the Benjamini and Hochberg [72] method.

## 3. Results

**Figure 1:**
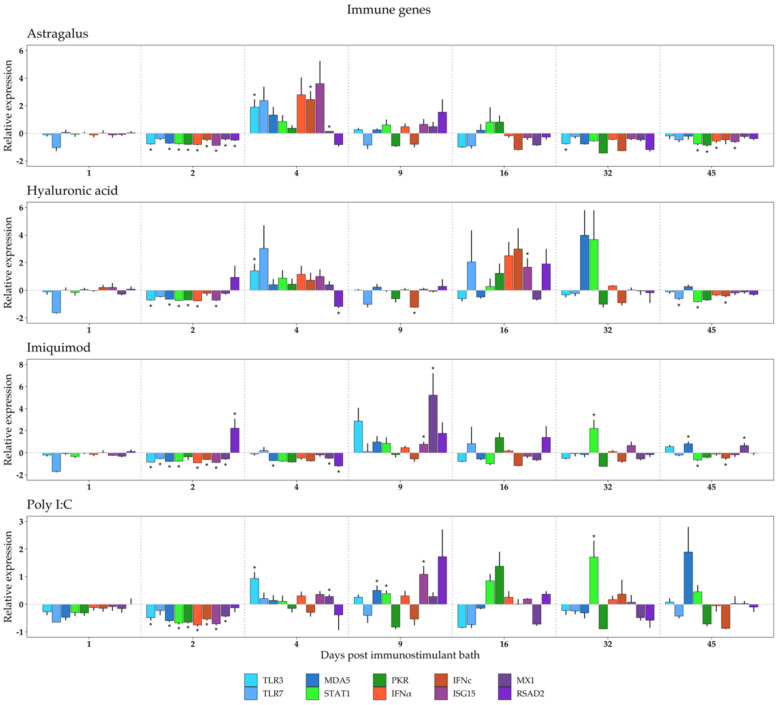
Relative expression of immune genes in Atlantic salmon larvae exposed to Astragalus, Hyaluronic acid, Imiquimod, or Poly I:C, sampled at 1, 2, 4, 9, 16, 32, and 45 days post immunostimulant bath. Transcription levels were assessed through qPCR and are presented as the mean ± standard error of mean gene expression relative to the expression of an HKG (18S). Different gene families are color coded in the following manner: blue—PRR; green—transcription factors; orange—interferons; purple—interferon-stimulated genes. Asterisks indicate *p* < 0.05 in a *t*-test. N = 6.

**Figure 2:**
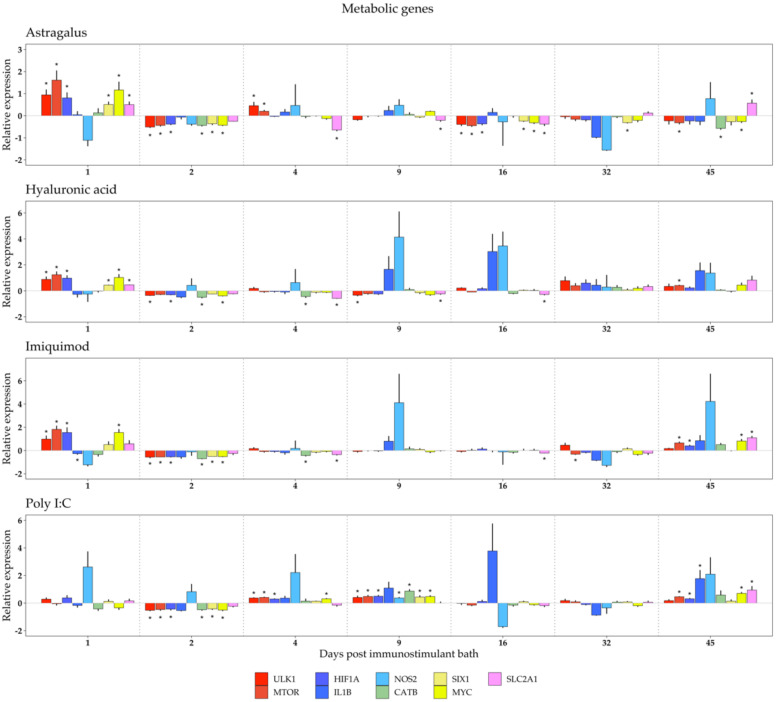
Relative expression of metabolic genes in Atlantic salmon larvae exposed to Astragalus, Hyaluronic acid, Imiquimod, or Poly I:C, sampled at 1, 2, 4, 9, 16, 32, and 45 days post immunostimulant bath. Transcription levels were assessed through qPCR and are presented as the mean ± standard error of mean gene expression relative to the expression of an HKG (18S). Different gene families are color coded in the following manner: red—regulation of autophagy; blue—cytokine production; green—Toll-like receptor signaling pathway; orange—transcription factors; pink—response to hypoxia. Asterisks indicate *p* < 0.05. N = 6.

Due to the importance of the innate immune system in antiviral response, it is hypothesized that stimulating disease resistance by upregulating the expression of key antiviral genes may be beneficial to afford increased protection during the delicate alevin stage. To be able to characterize the response to relevant immunostimulants, the relative expression of several immune- and metabolic-related genes were measured by qPCR. These data are presented in Figure 1 and Figure 2. Genes are grouped to facilitate result interpretation.

**Figure 3:**
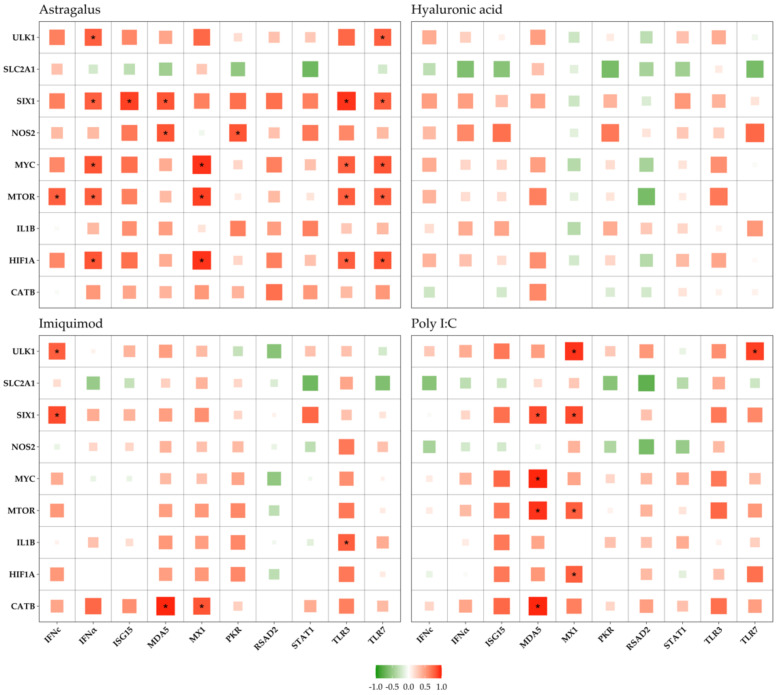
Spearman’s rank correlation between immune (x-axis) and metabolic genes (y-axis) in Astragalus, Hyaluronic acid, Imiquimod, and Poly I:C. Correlation varies between −1 (negatively correlated; green color) and 1 (positively correlated; red color). The size of the squares and depth of color indicate the strength of correlation. Asterisks indicate *p* < 0.05.

Additionally, to shed some light on the metabolic mechanisms underlying the immune response, correlations between gene groups were sought through Spearman’s rank correlation analysis, the results of which are presented in Figure 3.

### 3.1. Immune Response to Immunostimulant Bath

A generalized decrease in the relative expression of immune and metabolic genes was observed on days 1 and 2 post bath across all groups, which is likely related to a stress induction (see Section 2.1. Fish stock and experimental setup). All immunostimulants generated a response in immune gene expression (Figure 1) after this initial two-day decrease. In Astragalus-treated juveniles, this started on day 4 with the overexpression of PRRs (*TLR3*, *TLR7*, *MDA5*), transcription factors (*STAT1*, *PKR*), interferons (*IFNα*, *IFNc*), and one of the interferon-stimulated genes (*ISG15*) (Figure 1, first panel). Hyaluronic acid exposed larvae also exhibited a similar overexpression pattern on day 4, and again on day 16, when the expression of transcription factors, interferons, and two of the interferon-stimulated genes increased, possibly as a response to the upregulation of *TLR7* (Figure 1, second panel). On day 45, genes in both Astragalus and Hyaluronic acid groups were downregulated (Figure 1, first and second panels).

The immune response in Poly I:C appeared to be lower than in Astragalus, Hyaluronic acid, and Imiquimod (Figure 1, fourth panel). There was, however, a slight increase in *TLR3* expression on day 4, and in *ISG15* and RSAD2 on day 9. In the Imiquimod group, the gene upregulation was mainly seen on day 9, with the overexpression of *TLR3* and the three interferon-stimulated genes (*ISG15*, MX1, RSAD2) (Figure 1, third panel).

### 3.2. Metabolic Response to Immunostimulant Bath

Metabolic changes may likely occur due to cell activation during an immune response. By assessing the expression patterns of genes linked to cell metabolism (Figure 2), namely those involved in the regulation of autophagy (*MTOR* and *ULK1*), TLR signaling pathways (*CATB*), transcription factors (*SIX1, MYC*), cytokine production (*IL1B, NOS2,* and *HIF1A*), and organic anion transport (*SLC2A1*), it is possible to clarify which metabolic pathways are influenced by immunostimulation.

In line with the observations on the immune genes, there was an overall downregulation of gene expression of metabolic genes (in relation to the control group) on day 2 post immunostimulant bath. In the following sampling days, most genes did not appear particularly down- or upregulated across all immunostimulants, with absolute values of relative expression below 1. The exceptions were *IL1B* and *NOS2*, both of which were associated with cytokine production. These had mean relative expression above 1 on days 9, 16, and 45 in Hyaluronic acid (Figure 2; second panel), days 9 and 45 in Imiquimod (Figure 2; third panel), and day 45 in Poly I:C (Figure 2, fourth panel). On day 32, both genes were underexpressed in three immunostimulant groups, Astragalus, Imiquimod, and Poly I:C.

### 3.3. Immune vs. Metabolic Genes

A Spearman’s rank correlation test (Figure 3) was used with the intent of discerning a possible association between the expression of different genes; particularly, if there were any strong correlations between immune and metabolic genes. Several positive correlations were observed (red squares), meaning that the expression of the paired genes moves in the same direction (towards upregulation or downregulation). Comparatively, fewer negative correlations (green squares), or paired genes that varied in opposite directions (one upregulated, the other downregulated), were identified.

In Astragalus exposed alevins, (Figure 3; Astragalus) *HIF1A, MTOR, MYC*, and *SIX1* were significantly correlated (Spearman’s coefficient > 0.7; *p* < 0.05) with the TLRs, *MX1*, and *IFNα*. There were also strong significant correlations of *NOS2* with *MDA5* and *PKR*, and *ULK1* with *IFNα* and *TLR7*. In the Imiquimod group (Figure 3; Imiquimod), *CATB, IL1B, SIX1,* and *ULK1* were significantly correlated with several immune genes (Spearman’s coefficient > 0.7; *p* < 0.05). *CATB* correlated with *MDA5* and *MX1*, *SIX1* and *ULK1* correlated with *IFNc*, and *IL1B* correlated with *TLR3*. For Poly I:C (Figure 3; Poly I:C), immune gene *MDA5* correlated with *MTOR, SIX1, CATB,* and *MYC*, while *MX1* correlated with *MTOR, SIX1, HIF1A*, and *ULK1*. It is also interesting to point out that *SLC2A1* was negatively correlated, albeit not significantly, with most immune genes across all groups.

## 4. Discussion

### 4.1. Immune Genes

This study is the first to examine the presence and modulation of immune and metabolic gene expression in newly hatched salmon alevins. From our results, it is possible to affirm that alevins are fully equipped with the necessary antiviral and metabolic genes. In general, gene expression was downregulated on days 1 and 2 post bath, compared to untreated control alevins. We decided to leave control fish untreated by following the rationale that newly hatched alevins do not respond to stress before they are approximately three weeks old [73]. However, it appears there is a degree of response after bathing since most immune and metabolic gene expression was significantly downregulated on day 2. Whether this is due to handling stress or immunostimulant exposure is unknown. An additional control group with handling stress and bathing should have been included in the present experimental plan to determine whether the general downregulation was attributable to a stress response.

From our results, all immunostimulants elicited a response in immune gene expression, especially for alevins bathed in water containing Astragalus (Figure 1; first panel). In this treatment, upregulation of antiviral genes started on day 4 in a pattern that is consistent with an interferon-stimulated gene response. In a recent publication, Astragalus-fed zebrafish (*Danio rerio*) displayed higher protection against spring viremia of carp virus (SVCV) infections and expression of antiviral genes (type I IFN and MX genes) in the spleen than in a control group [74]. In line with the zebrafish work, elevated antiviral gene expression in turbot was found by feeding the fish with an experimental diet containing Astragalus [24]. In this study, the liver showed higher expression of Myeloid differentiation primary response 88 (MyD88) among other proinflammatory genes. MyD88 is the signaling adaptor of most TLR receptors but not TLR3, which has a TRAM/TRIF adaptor [75,76]. Concerning signaling, the Janus kinase–signal transducer and activator of transcription (JAK-STAT) pathway is responsible for the effective communication of signals from outside the cell to the nucleus [77]. JAK-STAT has been linked to cytokine signaling in Atlantic salmon [78], which correlates with the upregulation of *STAT1*.

Astragalus contains an array of different polysaccharides, saponins, and flavonoids [79]; thus, it is not clear which molecule(s) exert potential antiviral effects. It is an open question whether this overexpression, followed by downregulation at later time points, could make Astragalus an effective antiviral agent for use in commercial production of Atlantic salmon smolt. Astragalus is more researched in livestock species than in fish, where it has been shown, for example, to have an adjuvant effect in maternal broilers whose offspring chickens were immunized against H5N1 influenza [80], and also that it inhibits replication of porcine circovirus infection in vitro [39].

Hyaluronic acid, which can be released during sterile inflammation, may bind to TLR2 and TLR4 [81] and activate the transcription factor activator protein-1 (AP-1) downstream [82]. AP-1 is composed of a heterodimer belonging to the c-Fos, c-Jun, ATF, and JDP families [83]. AP-1 may be triggered by activation of both TLR2 and TLR4 [81], though salmon TLR4 does not seems to exist [84]. Ligand binding to TLR3 and TLR7 may also induce activation and nuclear translocation of AP-1 and subsequent gene expression [85].

Hyaluronic acid exposed larvae exhibited a similar overexpression pattern on day 4, and again on day 16 when especially the expression of *TLR3* and *ISG15* increased. However, a significant downregulation of *TLR7*, *STAT1,* and *IFNc* was observed on day 45. To the best of our knowledge, there is no other scholarly work that has examined the expression of antiviral genes after Hyaluronic acid treatment; thus, it is impossible to compare our results with prior studies.

Bathing of alevins in Imiquimod (mimic of viral ssRNA that binds TLR7) induced downregulation of *MDA5* expression on day 4. MDA5 is one member of the RLRs and is capable of recognizing dsRNA and Poly I:C. Perturbation of MDA5 may lead to the induction of type I IFNs, ISG15, and proinflammatory cytokines [86]. *MDA5* was found up-regulated on day 45. It is not clear how Imiquimod induced modulated expression of *MDA5* in the current study. An elevated *MDA5* kidney expression was observed when Imiquimod was injected in olive flounder (*Paralichthys olivaceus*) [87]. This supports our finding that *MDA5* expression may be modified by Imiquimod. *MX1* was upregulated on days 9 and 45, which is also in accordance with the results obtained by Avunje and Jung (2017). *STAT1* was upregulated on day 32 and downregulated on day 45.

Imiquimod is a known stimulant that induces the activation of the transcription factor STAT1, which may control type I IFN response and expression of ISGs [88,89]. In the current study, there was downregulation of both *STAT1* and *IFNc* on day 45 after bathing. A correlation between *STAT1* and *IFNc* has been observed in Nile tilapia (*Oreochromis niloticus*) which may support the dual downregulation found in our experiment [90].

The immune response in Poly I:C appeared to be generally weaker in terms of fold change gene expression than in alevins from the other immunostimulant groups. There was, however, a slight increase in *TLR3* and *MX1* expression on day 4, and for *ISG15*, *MDA5,* and *STAT1* on day 9. Poly I:C is a widely used TLR3 and MDA5 agonist (dsRNA mimic) in several fish species, including olive flounder [91], and has been shown to induce the expression of antiviral genes such as *ISG15* and *MX* in Atlantic salmon [92,93,94] and *STAT1* in vitro [95,96]. The current study confirms the upregulation of *ISG15* and *STAT1* by using Poly I:C.

### 4.2. Metabolic Genes

Contrary to what was observed in the immune genes, overall changes in metabolic gene expression in alevins were less pronounced when comparing treatment groups to control. An exception is for alevins bathed in Astragalus, in which the expression of *ULK1*, *MTOR, HIF1A, SIX1, MYC*, and *SLC2A1* was upregulated on day 1. Thereafter, metabolic gene expression was underexpressed on day 2 post bath, except for *IL1B* and *SLC2A1*, which were unaltered.

The findings from one-day-old alevins, which displayed increased expression of metabolic genes after bathing, may be due to handling the stress of having increased metabolic activity. An increase in metabolic activity has been shown before when trout alevins were stressed by acute hypoxia [97].

In addition, there was a notable increase in the expression of *MTOR*, *HIF1A*, *IL1B*, *MYC*, and *SLC2A1* on day 45 after bathing in water containing Poly I:C. The dependence of the mechanistic target of rapamycin (MTOR) on TLR3-induced cell activation has been shown in human keratinocytes [98]. Moreover, *HIF1A* has been found to be highly expressed during Poly I:C stimulation in mandarin fish (*Siniperca chuatsi*) [99]. *MYC* (and *HIF1A*) has previously been found to be highly expressed in human breast cancer cells treated with Poly I:C [100]. Lastly, expression of *SLC2A1* (*GLUT1*) has been shown to be induced in RAW macrophages stimulated by Poly I:C, indicative of increased glycolysis [101].

### 4.3. Gene Correlations

Despite the limited response seen in metabolic gene expression, the direction of change and correlation strength provide some insight into the interplay between immune and metabolic genes and, consequently, between cell metabolism and innate immunity.

It was in the Astragalus exposed alevins that the highest number of significant correlations between immune and metabolic genes was observed. Additionally, Imiquimod and Poly I:C groups also induced metabolic changes in terms of gene expression.

It is now well understood why immune cell activation requires cellular glucose metabolism to shift from oxidative phosphorylation to glycolysis as a way of making ATP readily available, a process first described in cancer cells (Warburg effect) [51,102]. To find out whether the immunostimulation of alevins really induced a Warburg effect, additional studies must be performed—such as lactate and ATP measurements, and determination of NAD+/NADH and NADP/NADPH ratios.

#### 4.3.1. Astragalus

MTOR coordinates eukaryotic cell growth and metabolism with environmental inputs including nutrients and growth factors. MTOR is a serine/threonine-protein kinase in the PI3K-related kinase (PIKK) family that forms the catalytic subunit of two distinct protein complexes, known as mTOR Complex 1 (mTORC1) and 2 (mTORC2). mTORC1 is sensitive to inhibition with rapamycin, and mTORC2 facilitates growth by promoting a shift in glucose metabolism from oxidative phosphorylation to glycolysis [103]. mTORC1 regulates cell growth since it has been shown that under nutrient-depleted conditions, mTORC1 phosphorylates ULK1, thereby preventing autophagy [104]. It has been shown that MTOR is able to regulate TLR3- and TLR7-mediated signaling in human keratinocytes [98,105]. In our study, we found a positive correlation between *MTOR* and *TLR3* and *TLR7* expression, which is in line with prior knowledge.

It is not clear how *MTOR* may regulate *MX1* expression, though a positive correlation between *MTOR* and *MX1* was found in this study. One possibility is that the alevins produced interferons, which, in turn, bind to the IFN receptor. The downstream cascade may promote the expression of *MX1* through the TYK2/JAK1 pathway [106]. *MTOR* is, similar to *TLR3* and *TLR7*, able to regulate IFN type I expression [107]. Our results show that there is a positive correlation between *MTOR* and *IFNα* (and *TLR3*, and *TLR7*) expression, which may be ascribed to the central regulatory role of *MTOR* in metabolism and immune gene expression.

Hypoxia-inducible factors (HIFs) are central regulators for cells to adapt to low cellular oxygen levels. HIF mediates the primary transcriptional response of a wide range of genes in response to hypoxia—such as regulating the transcription of a large array of genes involved in metabolism, cell survival, proliferation, migration, invasion, angiogenesis, immune evasion, and resistance to therapies in response to hypoxia [108]. HIF1A has been shown to inhibit the transcriptional activity of MYC as an adaptive response that promotes cell survival under low oxygen conditions [109]. Blocking MTOR function by rapamycin inhibits HIF1′s transcriptional activity [110]. This means that the higher transcriptional activity of HIF1A downregulates MYC’s ability to regulate cellular processes. The transcription factor MYC is a global regulator of gene expression involved in a myriad of responses, such as cellular division, differentiation, apoptosis, angiogenesis, DNA replication, RNA processing, metabolism, and ribosome biogenesis [108].

In a study using squamous cancer cells, it was shown that activation of TLR3 and TLR4 stimulated the expression of HIF-1 through NF-κB. In addition, HIF-1 increased the expression of *TLR3* and *TLR4* through direct promoter binding. Similar findings were reported in human fibroblasts; thus, the TLR/NF-κB pathway forms a positive feedback loop with HIF-1 [111,112]. In another study, it was indicated that TLR7 ligands (e.g., resiquimod—an analog of Imiquimod) induced HIF1A accumulation in a time- and concentration-dependent manner in THP-1 human leukemia monocytic macrophages [113]. However, the expression of *TLR7* remained unchanged during resiquimod stimulation. We found a positive correlation between *HIF1A* and *TLR7* expression after Astragalus bathing of alevins, whether this is a coincidence or not is not clear. This applies also to the present finding where there was a positive correlation between *MX1* and *HIF1A*. MX proteins belong to interferon-induced dynamin GTPase, which may inhibit virus replication. To the best of our knowledge, no research has been conducted to decipher the relationship between *HIF1A* and *MX1*. Furthermore, an interdependency between HIF1A and type I IFN has been shown in mouse embryonic fibroblasts [114], supporting our observation in which *HIF1A* expression was positively correlated with *IFNα* expression.

The expression of *ULK1* was positively correlated to the expression of *IFNα* and *TLR7*. The *ULK1* gene encodes the Unc-51-like autophagy kinase, which has several downstream phosphorylation targets during autophagosome formation and is activated during nutrient deprivation [115]. Functional ULK1 is required for gene transcription mediated via IFN-stimulated response elements (ISRE) and IFNγ activation site (GAS) elements and controls the expression of key ISGs [116]. It is known that *TLR7*-mediated cell activation induced autophagy [117], implying that there may exist a connection between *TLR7*, *IFNα,* and *ULK1*, as indicated by the concerted positive correlation between these three genes.

There were also positive correlations between *NOS2*, *MDA5,* and *PKR*. Inducible nitrogen synthase (NOS2/iNOS) may be produced by leukocytes in response to stimuli. Melanoma differentiation-associated protein 5 (MDA5) is an RLR for dsRNA. MDA5 has been shown to be induced by infectious bursal disease virus infection in chicken macrophages. Overexpression of *MDA5* with subsequent viral infection resulted in increased iNOS expression level [118]. Like MDA5, PKR (protein kinase R) is also able to bind dsRNA. Upon binding dsRNA, PKR undergoes autophosphorylation, activating itself. PKR (interferon inducible) then phosphorylates eIF2α, thus inhibiting protein synthesis in virally infected cells [119]. The MDA5/MAV5/TBK1/IKKe/IRF3/7 pathway induces, in turn, the expression of *iNOS* and *PKR*. Thus, based on available knowledge and compared to our results, it is likely that there is a connection between *NOS2*, *MDA5,* and *PKR*.

#### 4.3.2. Imiquimod

Imiquimod is an immune response modifier, acting as a TLR7 agonist. The expression of *MDA5* and *MX1* was positively correlated to the expression of *CATB, IL1B, SIX1,* and *ULK1*. It is apparent that Imiquimod can bind to MDA5, inducing type I IFN production that induces expression of ISGs—where *PKR*, *RSAD2,* and *MX1* are directly interfering with viral replication [120]. Transcription factor sine oculis homeobox 1 (SIX1) is a key regulator of organogenesis and is a central regulator of the Warburg effect that promotes glucose metabolism. However, a link between *SIX1, ULK1, CATB,* and *IL1B* with *MDA5* and *MX1* has not been reported before, and we are thus unsure whether this is a coincidence or not. As mentioned above, there is a link between *ULK1* and type I IFN expression [116], supporting the positive correlation between *ULK1* and *IFNc* in the current study. Engagement of the *TLR3* may bring about the expression of *IL1B*, as shown in another publication [121], which supports the present study. It is likely that *TLR3* may induce the expression of *IL1B* through the TICAM-1/TRIF pathway [122], but Imiquimod is not a TLR3 agonist. Thus, it is not clear why the expression of *TLR3* was positively correlated with *IL1B* expression.

#### 4.3.3. Poly I:C

*HIF1A, MYC, MTOR,* and *SIX1* are all recognized key regulators of the Warburg effect [123], while MDA5 senses dsRNA, associates to MAVS (adaptor protein IFNβ promoter stimulator 1 (IPS-1/MAVS)) which leads to the transcriptional activity of NF-kB and the induction of TBK1- and IRF-3-mediated type I IFN response [124]. It is not known whether MDA5 is involved in a Warburg-like response. In our study, there was a positive correlation between *HIF1A, MYC, MTOR, CATB* and *SIX1*, and *MDA5*, which may indicate an elevated metabolic modulation following Poly I:C stimulation of alevins. Cathepsin B (CATB), a lysosomal protease, is overexpressed in human breast cancers with high metabolic activity and is correlated with poor prognosis. Cathepsin B is a also regulator of metabolic processes [125]. It has been shown that HIF1A is involved in the modulated glycolytic activity. The expression of *HIF1A*, together with *MTOR* and *SIX1*, was positively correlated with *MX1* expression. We cannot explain how *MX1* RNA expression fits with increased gene expression of genes associated with metabolic activity. A plausible explanation is that treatment with Poly I:C both modulates the metabolic activity and induces *MX1* expression, as Poly I:C is a potent stimulator for *MX1* expression [93]. Upregulation of *MTOR, HIF1A*, and *MYC* have all been linked to this metabolic reprogramming that occurs upon PRR ligation in immune cells, promoting glycolysis by increasing the expression of *GLUT1* (*SLC2A1*; *glucose transporter 1*) and other glycolytic genes [43,48]. Consequently, we expected upregulation of *SLC2A1*, together with a positive correlation to PRR expression. Surprisingly, the opposite occurred throughout days 4 to 32, with downregulation of this gene when the immune response was highest in all immunostimulant groups. The expression of *SLC2A1* was negatively correlated with *PKR* and *RSAD2* (*viperin*) expression in our study. *PKR* has been shown to be activated by Poly I:C [126], similar to *RSAD2* and *HIF1A* [127,128]. We cannot provide a logical explanation for why all these three genes were collectively downregulated in this study.

## 5. Conclusions

Altogether, our results demonstrate that innate antiviral genes of Atlantic salmon are present at hatching, can be primed by exposure to immunostimulants, and possibly be functional to inhibit viral replication. Whether immunostimulation, by bathing, at early life stages translates into protection against viral diseases, thus making it an effective tool for use in commercial production of Atlantic salmon, remains to be proved. To the best of our knowledge, the concept of determining immune and metabolic gene response in tandem is not common. Our results highlight positive correlations between several immune and metabolic genes, underpinning that the bath treatments induced both innate and metabolic modulation. Further research should take a holistic approach to study the functional relationship between metabolism and innate antiviral defense mechanisms, which includes a pathogen challenge experiment.

## Figures and Tables

**Table 1:** Immunostimulants used in the present study, including concentration used, CAS number, and producer.

Immunostimulant	Concentration (mg L^−1^)	CAS No.	Producer
Astragalus root extract	10	89250-26-0	Beijing Solarbio Science & Technology Co. Ltd., Beijing, China
Hyaluronic acid	10	9004-61-9	Wuhan Yuancheng Gongchuang Technology Co. Ltd., Wuhan, China
Imiquimod	1	99011-02-6	Wuhan Yuancheng Gongchuang Technology Co. Ltd., Wuhan, China
Poly I:C	10	42424-50-0	Tianjin Kangtai Biotechnology Co. Ltd., Tianjin, China

**Table 2:** qPCR protocol for the Applied Biosystems 7500 Fast Real-Time PCR System. PCR product amplification was achieved through 40 cycles, followed by a melting curve stage to ensure reaction specificity.

	40 Cycles			Melting Curve Stage			
Temperature (°C)	95	95	60	95	60	95	60
Time (s)	20	3	60	15	60	15	15

**Table 3:** Selected genes and primer sequences. Gene symbols follow the HUGO Gene Nomenclature Committee approved nomenclature, except for *IFNα* and *IFNc*, which are fish specific. Where available, the gene symbol associated with the GenBank accession number is displayed between brackets.

Gene Symbol	Direction	Primer Sequence	GenBank Accession No.	Reference	Group
**18S**	Forward	TGTGCCGCTAGAGGTGAAATT	AJ427629.1	[53]	Housekeeping
	Reverse	GCAAATGCTTTCGCTTTCG			
**β-actin**	Forward	CAGCCCTCCTTCCTCGGTAT	BT059604	[54]	Housekeeping
	Reverse	CGTCACACTTCATGATGGAGTTG			
**EF1α**	Forward	CGCCAACATGGGCTGG	AN321836	[55]	Housekeeping
	Reverse	TCACACCATTGGCGTTACCA			
**NOS2** **(iNOS)**	Forward	AACGAGAGCCAACAGGTGTC	AJ300555.1	[56]	Metabolic
	Reverse	GGTGCAGCATGTCTTTGAGA			
**SLC2A1** **(GLUT1)**	Forward	CGCCAGCCCATCTTCATC	AF247728	[57]	Metabolic
	Reverse	GAAAACAGCGTTGATGCCAGA			
**MYC** **(c-Myc)**	Forward	TCTCCACCCACCAGCACAAC	DQ834862.1	Unpublished	Metabolic
	Reverse	CTCCAGCCTCAGCCTTTTGAC			
**MTOR** **(mTOR)**	Forward	CAGCCTGAGGCCCTGAATAA	BT072258	[58]	Metabolic
	Reverse	CTCCACTTGGGTTGGCACAT			
**ULK1**	Forward	CTAGCGTACATTGGGGCATT	XM014128422.1	Unpublished	Metabolic
	Reverse	CTTTCTCCTCCGTGAAGTCG			
**HIF1A** **(HIF-1α)**	Forward	CCACCTCATGAAGACCCATCA	DY708816	[58]	Metabolic
	Reverse	TCTCCACCCACACAAAGCCT			
**CATB** **(cathepsin B)**	Forward	AGGGGGGAACTCCTTACTGGCT	DR696159	[59]	Metabolic
	Reverse	CGATGCCACAGTGGTCCTTACCT			
**SIX1**	Forward	CCAGCTCGGAAGATGAGTTC	XM029676167.1	[57]	Metabolic
	Reverse	TAGAGGTCCCAGCAACGAGT			
**IL1B** **(IL-1β)**	Forward	GCTGGAGAGTGCTGTGGAAGA	AY617117	[57]	Metabolic
	Reverse	TGCTTCCCTCCTGCTCGTAG			
**IFNα**	Forward	TGCAGTATGCAGAGCGTGTG	DQ354152.1	[60]	Immune
	Reverse	TCTCCTCCCATCTGGTCCAG			
**IFNc**	Forward	ATGTATGATGGGCAGTGTGG	EU768890	[60]	Immune
	Reverse	CCAGGCGCAGTAACTGAAAT			
**ISG15**	Forward	CTGAAAAACGAAAAGGGCCA	AY926456.1	[60]	Immune
	Reverse	GCAGGGACTCCCTCCTTGTT			
**MDA5**	Forward	CTCGTGAACTACTCAAGAGAATCG	NM001195179	[61]	Immune
	Reverse	CCTGGCTCATCTATCAAGTTAT			
**MX1**	Forward	TGCAACCACAGAGGCTTTGAA	NM001123693.1	[60]	Immune
	Reverse	GGCTTGGTCAGGATGCCTAAT			
**PKR**	Forward	TGGCATGATGGAGACGACAG	EF523422.1	[60]	Immune
	Reverse	GCTGGGAGATAACTGCTCGG			
**STAT1**	Forward	GGTCCACACAAATCAACGTG	DW551983	[60]	Immune
	Reverse	CTTTGCAGGGCCTTCTTCTT			
**TLR3**	Forward	TTTGATGAGTCTCCGCCAACTCCA	BK008646	[60]	Immune
	Reverse	AATCTGCGAGGGACACAAAGGTCT			
**TLR7**	Forward	TACAGCTTGGTAACATGACTCTCC	AGKD01152847	[60]	Immune
	Reverse	CAACTCTCTGAGACTTGTCGGTAA			
**RSAD2** **(viperin)**	Forward	TCCTTGATGTTGGCGTGGAA	BT047610	[60]	Immune
	Reverse	GCATGTCAGCTTTGCTCCACA			

## Data Availability

The datasets generated for this article and the R code used have been made available at: https://github.com/UiT-RGG/figueiredo-et-al_2021 (accessed on 6 September 2021). Any extant queries concerning either the data or the code should be sent to filipe.figueiredo@uit.no.

## References

[1] FAO (2020). The State of World Fisheries and Aquaculture 2020, Sustainability in Action.

[2] Ingun S., Cecilie S., Walde J.B.B., Bornø G., Haukaas A., Brun E. (2020). The Health Situation in Norwegian Aquaculture 2019, Norwegian Veterinary Institute. https://www.vetinst.no/rapporter-og-publikasjoner/rapporter/2020/fish-health-report-2019/_/attachment/download/7507c5b2-df54-4028-aee8-da5ae3010d0d:728b5711c9fb60a0ba16803064780727f5bc3b6b/Fish%20health%20report%202019.pdf.

[3] Vadstein O., Bergh Ø., Gatesoupe F.-J., Galindo-Villegas J., Mulero V., Picchietti S., Scapigliati G., Makridis P., Olsen Y., Dierckens K. (2013). Microbiology and Immunology of Fish Larvae. Rev. Aquac..

[4] Aoki T., Hikima J., Hwang S.D., Jung T.S. (2013). Innate Immunity of Finfish: Primordial Conservation and Function of Viral RNA Sensors in Teleosts. Fish Shellfish Immunol..

[5] Janeway C.A. (1989). Approaching the Asymptote? Evolution and Revolution in Immunology. Cold Spring Harb. Symp. Quant. Biol..

[6] Murphy K., Weaver C. (2017). Janeway’s Immunobiology.

[7] Svingerud T., Holand J.K., Robertsen B. (2013). Infectious Salmon Anemia Virus (ISAV) Replication Is Transiently Inhibited by Atlantic Salmon Type I Interferon in Cell Culture. Virus Res..

[8] Beutler B., Jiang Z., Georgel P., Crozat K., Croker B., Rutschmann S., Du X., Hoebe K. (2006). Genetic Analysis of Host Resistance: Toll-like Receptor Signaling and Immunity at Large. Annu. Rev. Immunol..

[9] Chen S.N., Zou P.F., Nie P. (2017). Retinoic Acid-Inducible Gene I (RIG-I)-like Receptors (RLRs) in Fish: Current Knowledge and Future Perspectives. Immunology.

[10] Zhang L., Gao Z., Yu L., Zhang B., Wang J., Zhou J. (2018). Nucleotide-Binding and Oligomerization Domain (NOD)-like Receptors in Teleost Fish: Current Knowledge and Future Perspectives. J. Fish. Dis..

[11] Roy P., Panda S.P., Pal A., Jayasankar P., Das B.K. (2017). Ontogenetic Profile of Antiviral Mx Gene and Its Role in Innate Immunity in Mrigal, Cirrhinus Mrigala (Hamilton 1822). Aquac. Res..

[12] Vercammen E., Staal J., Beyaert R. (2008). Sensing of Viral Infection and Activation of Innate Immunity by Toll-like Receptor 3. Clin. Microbiol. Rev..

[13] Chu Q., Gao Y., Xu G., Wu C., Xu T. (2015). Transcriptome Comparative Analysis Revealed Poly(I:C) Activated RIG-I/MDA5-Mediated Signaling Pathway in Miiuy Croaker. Fish Shellfish Immunol..

[14] Kitao Y., Kono T., Korenaga H., Iizasa T., Nakamura K., Savan R., Sakai M. (2009). Characterization and Expression Analysis of Type I Interferon in Common Carp *Cyprinus carpio* L.. Mol. Immunol..

[15] Tanekhy M., Kono T., Sakai M. (2010). Cloning, Characterization, and Expression Analysis of Toll-like Receptor-7 CDNA from Common Carp, *Cyprinus carpio* L.. Comp. Biochem. Physiol. Part. D Genom. Proteom..

[16] Hemmi H., Kaisho T., Takeuchi O., Sato S., Sanjo H., Hoshino K., Horiuchi T., Tomizawa H., Takeda K., Akira S. (2002). Small-Antiviral Compounds Activate Immune Cells via the *TLR7* MyD88-Dependent Signaling Pathway. Nat. Immunol..

[17] Schön M.P., Schön M. (2007). Imiquimod: Mode of Action. Br. J. Dermatol..

[18] Flutter B., Nestle F.O. (2013). TLRs to Cytokines: Mechanistic Insights from the Imiquimod Mouse Model of Psoriasis. Eur. J. Immunol..

[19] Gong T., Liu L., Jiang W., Zhou R. (2020). DAMP-Sensing Receptors in Sterile Inflammation and Inflammatory Diseases. Nat. Rev. Immunol..

[20] Zhang J., Wang H., Xiao Q., Liang H., Li Z., Jiang C., Wu H., Zheng Q. (2009). Hyaluronic Acid Fragments Evoke Kupffer Cells via TLR4 Signaling Pathway. Sci. China C Life Sci. C.

[21] You N., Chu S., Cai B., Gao Y., Hui M., Zhu J., Wang M. (2021). Bioactive Hyaluronic Acid Fragments Inhibit Lipopolysaccharide-Induced Inflammatory Responses via the Toll-like Receptor 4 Signaling Pathway. Front. Med..

[22] Senbanjo L.T., Chellaiah M.A. (2017). CD44: A Multifunctional Cell Surface Adhesion Receptor Is a Regulator of Progression and Metastasis of Cancer Cells. Front. Cell Dev. Biol..

[23] Cermelli C., Cuoghi A., Scuri M., Bettua C., Neglia R.G., Ardizzoni A., Blasi E., Iannitti T., Palmieri B. (2011). In Vitro Evaluation of Antiviral and Virucidal Activity of a High Molecular Weight Hyaluronic Acid. Virol. J..

[24] Sun Y., Wang X., Zhou H., Mai K., He G. (2020). Dietary Astragalus Polysaccharides Ameliorates the Growth Performance, Antioxidant Capacity and Immune Responses in Turbot (*Scophthalmus maximus* L.). Fish Shellfish. Immunol..

[25] Jian J., Wu Z. (2003). Effects of Traditional Chinese Medicine on Nonspecific Immunity and Disease Resistance of Large Yellow Croaker, *Pseudosciaena crocea* (Richardson). Aquaculture.

[26] Liu Y., Miao Y., Xu N., Ding T., Cui K., Chen Q., Zhang J., Fang W., Mai K., Ai Q. (2020). Effects of Dietary Astragalus Polysaccharides (APS) on Survival, Growth Performance, Activities of Digestive Enzyme, Antioxidant Responses and Intestinal Development of Large Yellow Croaker (*Larimichthys crocea*) Larvae. Aquaculture.

[27] Ardó L., Yin G., Xu P., Váradi L., Szigeti G., Jeney Z., Jeney G. (2008). Chinese Herbs (*Astragalus membranaceus* and *Lonicera japonica*) and Boron Enhance the Non-Specific Immune Response of Nile Tilapia (*Oreochromis niloticus*) and Resistance against Aeromonas Hydrophila. Aquaculture.

[28] Elabd H., Wang H.P., Shaheen A., Matter A. (2020). *Astragalus membranaceus* Nanoparticles Markedly Improve Immune and Anti-Oxidative Responses; and Protection against *Aeromonas veronii* in Nile Tilapia *Oreochromis niloticus*. Fish Shellfish Immunol..

[29] Tang J., Cai J., Liu R., Wang J., Lu Y., Wu Z., Jian J. (2014). Immunostimulatory Effects of Artificial Feed Supplemented with a Chinese Herbal Mixture on *Oreochromis niloticus* against *Aeromonas hydrophila*. Fish Shellfish Immunol..

[30] Zahran E., Risha E., AbdelHamid F., Mahgoub H.A., Ibrahim T. (2014). Effects of Dietary Astragalus Polysaccharides (APS) on Growth Performance, Immunological Parameters, Digestive Enzymes, and Intestinal Morphology of Nile Tilapia (*Oreochromis niloticus*). Fish Shellfish Immunol..

[31] Lin S.M., Jiang Y., Chen Y.J., Luo L., Doolgindachbaporn S., Yuangsoi B. (2017). Effects of Astragalus Polysaccharides (APS) and Chitooligosaccharides (COS) on Growth, Immune Response and Disease Resistance of Juvenile Largemouth Bass, Micropterus Salmoides. Fish Shellfish Immunol..

[32] Mo W.Y., Lun C.H.I., Choi W.M., Man Y.B., Wong M.H. (2016). Enhancing Growth and Non-Specific Immunity of Grass Carp and Nile Tilapia by Incorporating Chinese Herbs (*Astragalus membranaceus* and *Lycium barbarum*) into Food Waste Based Pellets. Environ. Pollut..

[33] Yuan C., Pan X., Gong Y., Xia A., Wu G., Tang J., Han X. (2008). Effects of Astragalus Polysaccharides (APS) on the Expression of Immune Response Genes in Head Kidney, Gill and Spleen of the Common Carp, *Cyprinus carpio* L.. Int. Immunopharmacol..

[34] Elabd H., Wang H.P., Shaheen A., Yao H., Abbass A. (2016). Feeding *Glycyrrhiza glabra* (Liquorice) and *Astragalus membranaceus* (AM) Alters Innate Immune and Physiological Responses in Yellow Perch (*Perca flavescens*). Fish Shellfish Immunol..

[35] Shi H., Yu F., Wang Q., Zhang T.-C., Nakajima M. (2015). Astragalus Membranaceus Polysaccharide-Enhanced Lymphocytes Proliferation of Yellow Drum Nibea albiflora In Vitro. Proceedings of the Advances in Applied Biotechnology.

[36] Bai D., Wu X., Zhu G., Guo Y., Yang G., Ning B., Xing K. (2012). Astragalus Polysaccharides Enhance Cellular Immune Response and Disease Resistance in Yellow Catfish. Isr. J. Aquac..

[37] Jian J., Wu Z. (2004). Influences of Traditional Chinese Medicine on Non-Specific Immunity of Jian Carp (*Cyprinus carpio Var. Jian*). Fish Shellfish Immunol..

[38] Zhang P., Liu X., Liu H., Wang W., Liu X., Li X., Wu X. (2018). Astragalus Polysaccharides Inhibit Avian Infectious Bronchitis Virus Infection by Regulating Viral Replication. Microb. Pathog..

[39] Xue H., Gan F., Zhang Z., Hu J., Chen X., Huang K. (2015). Astragalus Polysaccharides Inhibits PCV2 Replication by Inhibiting Oxidative Stress and Blocking NF-ΚB Pathway. Int. J. Biol. Macromol..

[40] Shao B.-M., Xu W., Dai H., Tu P., Li Z., Gao X.-M. (2004). A Study on the Immune Receptors for Polysaccharides from the Roots of *Astragalus membranaceus*, a Chinese Medicinal Herb. Biochem. Biophys. Res. Commun..

[41] Zhou L., Liu Z., Wang Z., Yu S., Long T., Zhou X., Bao Y. (2017). Astragalus Polysaccharides Exerts Immunomodulatory Effects via TLR4-Mediated MyD88-Dependent Signaling Pathway In Vitro and In Vivo. Sci. Rep..

[42] Wei W., Xiao H.-T., Bao W.-R., Ma D.-L., Leung C.-H., Han X.-Q., Ko C.-H., Lau C.B.-S., Wong C.-K., Fung K.-P. (2016). TLR-4 May Mediate Signaling Pathways of Astragalus Polysaccharide RAP Induced Cytokine Expression of RAW264.7 Cells. J. Ethnopharmacol..

[43] Corcoran S.E., O’Neill L.A.J. (2016). HIF1α and Metabolic Reprogramming in Inflammation. J. Clin. Investig..

[44] Fitzgerald K.A., Kagan J.C. (2020). Toll-like Receptors and the Control of Immunity. Cell.

[45] Ganeshan K., Chawla A. (2014). Metabolic Regulation of Immune Responses. Annu. Rev. Immunol..

[46] O’Neill L.A.J., Kishton R.J., Rathmell J. (2016). A Guide to Immunometabolism for Immunologists. Nat. Rev. Immunol..

[47] Pearce E.L., Pearce E.J. (2013). Metabolic Pathways in Immune Cell Activation and Quiescence. Immunity.

[48] Weichhart T., Hengstschläger M., Linke M. (2015). Regulation of Innate Immune Cell Function by MTOR. Nat. Rev. Immunol..

[49] Grodzki A.C.G., Giulivi C., Lein P.J. (2013). Oxygen Tension Modulates Differentiation and Primary Macrophage Functions in the Human Monocytic THP-1 Cell Line. PLoS ONE.

[50] Langston P.K., Shibata M., Horng T. (2017). Metabolism Supports Macrophage Activation. Front. Immunol..

[51] O’Neill L.A.J., Pearce E.J. (2015). Immunometabolism Governs Dendritic Cell and Macrophage Function. J. Exp. Med..

[52] Liu Y., Xin Z.Z., Zhang D.Z., Wang Z.F., Zhu X.Y., Tang B.P., Jiang S.H., Zhang H.B., Zhou C.L., Chai X.Y. (2017). Transcriptome Analysis of Yellow Catfish (*Pelteobagrus fulvidraco*) Liver Challenged with Polyriboinosinic Polyribocytidylic Acid (Poly I:C). Fish Shellfish Immunol..

[53] Soleng M., Johansen L.-H., Johnsen H., Johansson G.S., Breiland M.W., Rørmark L., Pittman K., Pedersen L.-F., Lazado C.C. (2019). Atlantic Salmon (*Salmo Salar*) Mounts Systemic and Mucosal Stress Responses to Peracetic Acid. Fish Shellfish Immunol..

[54] Jensen I., Overrein M.C., Fredriksen B.N., Strandskog G., Seternes T. (2019). Differences in Smolt Status Affect the Resistance of Atlantic Salmon (*Salmo salar* L.) against Infectious Pancreatic Necrosis, While Vaccine-Mediated Protection Is Unaffected. J. Fish Dis..

[55] Johansen L.-H., Dahle M.K., Wessel Ø., Timmerhaus G., Løvoll M., Røsæg M., Jørgensen S.M., Rimstad E., Krasnov A. (2016). Differences in Gene Expression in Atlantic Salmon Parr and Smolt after Challenge with *Piscine orthoreovirus* (PRV). Mol. Immunol..

[56] Braden L.M., Barker D.E., Koop B.F., Jones S.R.M. (2012). Comparative Defense-Associated Responses in Salmon Skin Elicited by the Ectoparasite *Lepeophtheirus salmonis*. Comp. Biochem. Physiol. Part D Genom. Proteom..

[57] Ulvestad J.S., Kumari J., Seternes T., Chi H., Dalmo R.A. (2018). Studies on the Effects of LPS, ß-Glucan and Metabolic Inhibitors on the Respiratory Burst and Gene Expression in Atlantic Salmon Macrophages. J. Fish. Dis..

[58] Olsvik P.A., Vikeså V., Lie K.K., Hevrøy E.M. (2013). Transcriptional Responses to Temperature and Low Oxygen Stress in Atlantic Salmon Studied with Next-Generation Sequencing Technology. BMC Genom..

[59] Bahuaud D., Mørkøre T., Langsrud Ø., Sinnes K., Veiseth E., Ofstad R., Thomassen M.S. (2008). Effects of −1.5 °C Super-Chilling on Quality of Atlantic Salmon (*Salmo salar*) Pre-Rigor Fillets: Cathepsin Activity, Muscle Histology, Texture and Liquid Leakage. Food Chem..

[60] Svingerud T., Solstad T., Sun B., Nyrud M.L.J., Kileng Ø., Greiner-Tollersrud L., Robertsen B. (2012). Atlantic Salmon Type I IFN Subtypes Show Differences in Antiviral Activity and Cell-Dependent Expression: Evidence for High IFNb/*IFNc*–Producing Cells in Fish Lymphoid Tissues. J. Immunol..

[61] Moore L.J., Jarungsriapisit J., Nilsen T.O., Stefansson S., Taranger G.L., Secombes C.J., Morton H.C., Patel S. (2017). Immune Gene Profiles in Atlantic Salmon (*Salmo Salar* L.) Post-Smolts Infected with SAV3 by Bath-Challenge Show a Delayed Response and Lower Levels of Gene Transcription Compared to Injected Fish. Fish Shellfish Immunol..

[62] Pfaffl M.W. (2001). A New Mathematical Model for Relative Quantification in Real-Time RT-PCR. Nucleic Acids Res..

[63] Kassambara A. (2020). ggpubr: “ggplot2” Based Publication Ready Plots. https://CRAN.R-project.org/package=ggpubr.

[64] R Core Team A Language and Environment for Statistical Computing (2020). R Foundation for Statistical Computing.

[65] Harrell F.E. (2021). Hmisc: Harrell Miscellaneous. https://CRAN.R-project.org/package=Hmisc.

[66] Cooper N. (2018). NCmisc: Miscellaneous Functions for Creating Adaptive Functions and Scripts. https://search.r-project.org/CRAN/refmans/NCmisc/html/00Index.html.

[67] Waddell J. (2015). oaColors: OpenAnalytics Colors Package. https://CRAN.R-project.org/package=oaColors.

[68] Pedersen T.L. (2020). patchwork: The Composer of Plots. https://cran.r-project.org/package=patchwork.

[69] Wickham H., Bryan J. (2019). readxl: Read. https://CRAN.R-project.org/package=readxl.

[70] Kassambara A. (2021). rstatix: Pipe-Friendly Framework for Basic Statistical Tests. https://cran.r-project.org/package=rstatix.

[71] Wickham H., Averick M., Bryan J., Chang W., McGowan L.D., François R., Grolemund G., Hayes A., Henry L., Hester J. (2019). Welcome to the Tidyverse. J. Open Source Softw..

[72] Benjamini Y., Hochberg Y. (1995). Controlling the False Discovery Rate: A Practical and Powerful Approach to Multiple Testing. J. R. Stat. Soc. Ser. B Stat. Methodol..

[73] Pottinger T.G., Mosuwe E. (1994). The Corticosteroidogenic Response of Brown and Rainbow Trout Alevins and Fry to Environmental Stress during a “Critical Period. ” Gen. Comp. Endocrinol..

[74] Li Y., Ran C., Wei K., Xie Y., Xie M., Zhou W., Yang Y., Zhang Z., Lv H., Ma X. (2021). The Effect of Astragalus Polysaccharide on Growth, Gut and Liver Health, and Anti-Viral Immunity of Zebrafish. Aquaculture.

[75] Kumar H., Kawai T., Akira S. (2011). Pathogen Recognition by the Innate Immune System. Int. Rev. Immunol..

[76] Li Y., Li Y., Cao X., Jin X., Jin T. (2017). Pattern Recognition Receptors in Zebrafish Provide Functional and Evolutionary Insight into Innate Immune Signaling Pathways. Cell Mol. Immunol..

[77] Renauld J.-C. (2003). Class II Cytokine Receptors and Their Ligands: Key Antiviral and Inflammatory Modulators. Nat. Rev. Immunol..

[78] Skjesol A., Hansen T., Shi C.-Y., Thim H.L., Jørgensen J.B. (2010). Structural and Functional Studies of *STAT1* from Atlantic Salmon (*Salmo salar*). BMC Immunol..

[79] Zhang J., Xie X., Li C., Fu P. (2009). Systematic Review of the Renal Protective Effect of *Astragalus Membranaceus* (Root) on Diabetic Nephropathy in Animal Models. J. Ethnopharmacol..

[80] Abdullahi A.Y., Kallon S., Yu X., Zhang Y., Li G. (2016). Vaccination with Astragalus and Ginseng Polysaccharides Improves Immune Response of Chickens against H5N1 Avian Influenza Virus. Biomed. Res. Int..

[81] Stenson W.F., Ciorba M.A. (2020). Nonmicrobial Activation of TLRs Controls Intestinal Growth, Wound Repair, and Radioprotection. Front. Immunol..

[82] Li N., Geng C., Hou S., Fan H., Gong Y. (2020). Damage-Associated Molecular Patterns and Their Signaling Pathways in Primary Blast Lung Injury: New Research Progress and Future Directions. Int. J. Mol. Sci..

[83] Eckert R.L., Adhikary G., Young C.A., Jans R., Crish J.F., Xu W., Rorke E.A. (2013). AP1 Transcription Factors in Epidermal Differentiation and Skin Cancer. J. Skin Cancer.

[84] Palti Y. (2011). Toll-like Receptors in Bony Fish: From Genomics to Function. Dev. Comp. Immunol..

[85] Jensen S., Thomsen A.R. (2012). Sensing of RNA Viruses: A Review of Innate Immune Receptors Involved in Recognizing RNA Virus Invasion. J. Virol..

[86] Lazarte J.M.S., Thompson K.D., Jung T.S. (2019). Pattern Recognition by Melanoma Differentiation-Associated Gene 5 (*MDA5*) in Teleost Fish: A Review. Front. Immunol..

[87] Avunje S., Jung S.-J. (2017). Poly (I:C) and Imiquimod Induced Immune Responses and Their Effects on the Survival of Olive Flounder (*Paralichthys olivaceus*) from Viral Haemorrhagic Septicaemia. Fish Shellfish Immunol..

[88] Bottrel R.L.A., Yang Y.-L., Levy D.E., Tomai M., Reis L.F.L. (1999). The Immune Response Modifier Imiquimod Requires STAT-1 for Induction of Interferon, Interferon-Stimulated Genes, and Interleukin-6. Antimicrob. Agents Chemother..

[89] Yu F.-F., Zhang Y.-B., Liu T.-K., Liu Y., Sun F., Jiang J., Gui J.-F. (2010). Fish Virus-Induced Interferon Exerts Antiviral Function through *STAT1* Pathway. Mol. Immunol..

[90] Gan Z., Cheng J., Chen S., Laghari Z.A., Hou J., Xia L., Lu Y., Nie P. (2020). Functional Characterization of a Group II Interferon, *IFNc* in the Perciform Fish, Nile Tilapia (*Oreochromis niloticus*). Fish Shellfish Immunol..

[91] Zhou Z., Zhang B., Sun L. (2014). Poly(I:C) Induces Antiviral Immune Responses in Japanese Flounder (Paralichthys Olivaceus) That Require *TLR3* and *MDA5* and Is Negatively Regulated by Myd88. PLoS ONE.

[92] Dixit E., Kagan J.C., Alt F.W. (2013). Chapter Four—Intracellular Pathogen Detection by RIG-I-Like Receptors. Advances in Immunology.

[93] Jensen I., Albuquerque A., Sommer A.-I., Robertsen B. (2002). Effect of Poly I:C on the Expression of Mx Proteins and Resistance against Infection by Infectious Salmon Anaemia Virus in Atlantic Salmon. Fish Shellfish Immunol..

[94] Kileng Ø., Albuquerque A., Robertsen B. (2008). Induction of Interferon System Genes in Atlantic Salmon by the Imidazoquinoline S-27609, a Ligand for Toll-like Receptor 7. Fish Shellfish Immunol..

[95] Chen A., Diaz-Soto M.P., Sanmamed M.F., Adams T., Schupp J.C., Gupta A., Britto C., Sauler M., Yan X., Liu Q. (2021). Single-Cell Characterization of a Model of Poly I:C-Stimulated Peripheral Blood Mononuclear Cells in Severe Asthma. Respir. Res..

[96] Dempoya J., Matsumiya T., Imaizumi T., Hayakari R., Xing F., Yoshida H., Okumura K., Satoh K. (2012). Double-Stranded RNA Induces Biphasic *STAT1* Phosphorylation by Both Type I Interferon (IFN)-Dependent and Type I IFN-Independent Pathways. J. Virol..

[97] Polymeropoulos E.T., Elliott N.G., Frappell P.B. (2019). Acute but Not Chronic Hyperoxia Increases Metabolic Rate without Altering the Cardiorespiratory Response in Atlantic Salmon Alevins. Aquaculture.

[98] Zhao J., Benakanakere M.R., Hosur K.B., Galicia J.C., Martin M., Kinane D.F. (2010). Mammalian Target of Rapamycin (MTOR) Regulates TLR3 Induced Cytokines in Human Oral Keratinocytes. Mol. Immunol..

[99] He J., Yu Y., Qin X.-W., Zeng R.-Y., Wang Y.-Y., Li Z.-M., Mi S., Weng S.-P., Guo C.-J., He J.-G. (2019). Identification and Functional Analysis of the Mandarin Fish (*Siniperca chuatsi*) Hypoxia-Inducible Factor-1α Involved in the Immune Response. Fish Shellfish Immunol..

[100] Scatozza F., D’Amore A., Fontanella R.A., DE Cesaris P., Marampon F., Padula F., Ziparo E., Riccioli A., Filippini A. (2020). Toll-Iike Receptor-3 Activation Enhances Malignant Traits in Human Breast Cancer Cells Through Hypoxia-Inducible Factor-1α. Anticancer Res..

[101] Feingold K.R., Shigenaga J.K., Kazemi M.R., McDonald C.M., Patzek S.M., Cross A.S., Moser A., Grunfeld C. (2012). Mechanisms of Triglyceride Accumulation in Activated Macrophages. J. Leukoc. Biol..

[102] Liang S., Ji L., Kang L., Hu X., Dong C., Jiang Z. (2020). Chapter Five—Metabolic regulation of innate immunity. Advances in Immunology in China—Part B.

[103] Saxton R.A., Sabatini D.M. (2017). MTOR Signaling in Growth, Metabolism, and Disease. Cell.

[104] Kim J., Kundu M., Viollet B., Guan K.-L. (2011). AMPK and MTOR Regulate Autophagy through Direct Phosphorylation of Ulk1. Nat. Cell Biol..

[105] Buechler M.B., Akilesh H.M., Hamerman J.A. (2016). Cutting Edge: Direct Sensing of *TLR7* Ligands and Type I IFN by the Common Myeloid Progenitor Promotes MTOR/PI3K-Dependent Emergency Myelopoiesis. J. Immunol..

[106] Wang B.X., Fish E.N. (2012). The Yin and Yang of Viruses and Interferons. Trends Immunol..

[107] Katholnig K., Linke M., Pham H., Hengstschläger M., Weichhart T. (2013). Immune Responses of Macrophages and Dendritic Cells Regulated by MTOR Signaling. Biochem. Soc. Trans..

[108] Li Y., Sun X.-X., Qian D.Z., Dai M.-S. (2020). Molecular Crosstalk Between MYC and HIF in Cancer. Front. Cell Dev. Biol..

[109] Gordan J.D., Bertout J.A., Hu C.-J., Diehl J.A., Simon M.C. (2007). HIF-2alpha Promotes Hypoxic Cell Proliferation by Enhancing c-Myc Transcriptional Activity. Cancer Cell.

[110] Wei J., Jiang H., Gao H., Wang G. (2016). Blocking Mammalian Target of Rapamycin (MTOR) Attenuates HIF-1α Pathways Engaged-Vascular Endothelial Growth Factor (VEGF) in Diabetic Retinopathy. Cell Physiol. Biochem..

[111] Han S., Xu W., Wang Z., Qi X., Wang Y., Ni Y., Shen H., Hu Q., Han W. (2016). Crosstalk between the HIF-1 and Toll-like Receptor/Nuclear Factor-ΚB Pathways in the Oral Squamous Cell Carcinoma Microenvironment. Oncotarget.

[112] Liu C., Ruan H., Himmati F., Zhao M.-T., Chen C.C., Makar M., Chen I.Y., Sallam K., Mocarski E.S., Sayed D. (2020). HIF1α Regulates Early Metabolic Changes Due to Activation of Innate Immunity in Nuclear Reprogramming. Stem Cell Rep..

[113] Nicholas S.A., Sumbayev V.V. (2009). The Involvement of Hypoxia-Inducible Factor 1 Alpha in Toll-like Receptor 7/8-Mediated Inflammatory Response. Cell Res..

[114] López D.M., Mesri E., Andreanksy S. (2020). Activation of the Hypoxia-Inducible Factor 1 Alpha Is Necessary for Type 1 Interferon and IFN Stimulatory Gene Expression during Gammaherpesvirus Infection. J. Immunol..

[115] Mizushima N. (2010). The Role of the Atg1/ULK1 Complex in Autophagy Regulation. Curr. Opin. Cell Biol..

[116] Saleiro D., Mehrotra S., Kroczynska B., Beauchamp E.M., Lisowski P., Majchrzak-Kita B., Bhagat T.D., Stein B.L., McMahon B., Altman J.K. (2015). Central Role of ULK1 in Type I Interferon Signaling. Cell Rep..

[117] Delgado M.A., Elmaoued R.A., Davis A.S., Kyei G., Deretic V. (2008). Toll-like Receptors Control Autophagy. EMBO J..

[118] Lee C.-C., Wu C.C., Lin T.L. (2015). Role of Chicken Melanoma Differentiation-Associated Gene 5 in Induction and Activation of Innate and Adaptive Immune Responses to Infectious Bursal Disease Virus in Cultured Macrophages. Arch. Virol..

[119] Lemaire P.A., Anderson E., Lary J., Cole J.L. (2008). Mechanism of *PKR* Activation by DsRNA. J. Mol. Biol..

[120] Verrier E.R., Langevin C., Benmansour A., Boudinot P. (2011). Early Antiviral Response and Virus-Induced Genes in Fish. Dev. Comp. Immunol..

[121] Lee H.J., Choi B., Kim Y., Lee S.E., Jin H.J., Lee H.-S., Chang E.-J., Kim S.W. (2019). The Upregulation of Toll-Like Receptor 3 via Autocrine IFN-β Signaling Drives the Senescence of Human Umbilical Cord Blood-Derived Mesenchymal Stem Cells Through JAK1. Front. Immunol..

[122] Kawasaki T., Kawai T., Vanpouille-Box C., Galluzzi L. (2019). Chapter One—Discrimination Between Self and Non-Self-Nucleic Acids by the Innate Immune System. Nucleic Acid Sensing and Immunity, Part A.

[123] Li L., Liang Y., Kang L., Liu Y., Gao S., Chen S., Li Y., You W., Dong Q., Hong T. (2018). Transcriptional Regulation of the Warburg Effect in Cancer by SIX1. Cancer Cell.

[124] Schenten D., Medzhitov R., Alt F.W. (2011). Chapter 3—The Control of Adaptive Immune Responses by the Innate Immune System. Advances in Immunology.

[125] Weiss-Sadan T., Itzhak G., Kaschani F., Yu Z., Mahameed M., Anaki A., Ben-Nun Y., Merquiol E., Tirosh B., Kessler B. (2019). Cathepsin L Regulates Metabolic Networks Controlling Rapid Cell Growth and Proliferation. Mol. Cell Proteomics.

[126] Gal-Ben-Ari S., Barrera I., Ehrlich M., Rosenblum K. (2019). *PKR*: A Kinase to Remember. Front. Mol. Neurosci..

[127] Wang B., Zhang Y.-B., Liu T.-K., Shi J., Sun F., Gui J.-F. (2014). Fish Viperin Exerts a Conserved Antiviral Function through RLR-Triggered IFN Signaling Pathway. Dev. Comp. Immunol..

[128] Hong S.W., Yoo J.W., Kang H.S., Kim S., Lee D.-K. (2009). HIF-1alpha-Dependent Gene Expression Program during the Nucleic Acid-Triggered Antiviral Innate Immune Responses. Mol. Cells.

